# The lipid card in cardiovascular patients: early enthusiasm but limited long-term use

**DOI:** 10.1007/s00392-025-02772-8

**Published:** 2025-10-15

**Authors:** Mohamad Amer Nashtar, Ali Canbay, Polykarpos Christos Patsalis, Martin Steinmetz

**Affiliations:** 1 Department of Internal Medicine, Division of Cardiology, Angiology and Internal Emergency Medicine, Knappschaft Kliniken University Hospital Bochum, Bochum, Germany; 2 Department of Internal Medicine, Division of Hepatology and Gastroenterology, Knappschaft Kliniken University Hospital Bochum, Bochum, Germany

Sirs,

Despite the substantial promise of a lipidcard—akin to Germany’s initiative within the “Auf Ziel 55” campaign—to engage primary care providers (PCPs) and empower patients, and very successful early study data from “Jena auf Ziel 55” among patients with ST-elevation myocardial infarction (STEMI) [1], real-world data reveal a troubling gap between early enthusiasm and sustained impact. In an effort to improve lipid monitoring and patient engagement, we introduced a lipid card in 2024 to 376 patients with established cardiovascular disease at our center (94% coronary artery disease, 2% peripheral artery disease, 4% carotid artery stenosis > 50%). The mean age was 66.3 ± 15 years, and 71% were male. Patients received the card during a structured consultation with medical counseling on lipid targets and cardiovascular risk management. Upon receiving the lipid card, patients were asked to complete a questionnaire assessing its usage and perceived utility; a follow-up questionnaire was administered 6 months later (*n* = 230, 62%).

*The promise of lipid cards—*Targeting LDL-C levels < 55 mg/dL, in keeping with ESC/EAS 2019 guidelines, is essential for very high-risk populations [2]. The DA VINCI study found only 18% of patients with established atherosclerotic cardiovascular disease on guideline—recommended therapy achieved LDL-C < 1.4 mmol/L (≈55 mg/dL); even with high-intensity statins or ezetimibe, goal attainment rose to just 37% [3]. Similar findings in Central and Eastern Europe showed that nearly three-quarters failed to reach 2019 LDL-C targets [4]. Given these sobering results, tools that enhance communication between patient and provider—such as structured lipid cards—appear all the more necessary.

*Uptake: encouraging but fleeting—*Early pilot data were encouraging: approximately 75% of patients receiving lipid cards—in our case both patients with acute coronary syndromes (42%: 24% STEMI, 33% NSTEMI, 57% unstable angina pectoris) and a slightly bigger proportion of chronic coronary syndromes (58%)—reported finding it helpful to be better informed about their health, felt that it would improve communication with their doctor, and expressed intent to use it consistently. However, this enthusiasm substantially waned, with only ~ 25% still using or referencing it at 6 months. Only 18% found that it improved communication with their physician. Why the drop? Our experience highlights a core barrier: PCPs rarely engage with or review thecards. With typical visits lasting just 3–5 min, PCPs seldom get to the card—even when patients bring it to consultations. Forty-six percent of respondents reported that their PCPs had declined to complete or review the lipid card at least once.

*Structural barriers in primary care*—Primary care workflows are tightly scheduled. PCPs juggle medication reviews, comorbidities, and acute concerns, often leaving little room for the additional administrative step of reviewing a lipid card. A 2014 Italian study found that GP-focused educational interventions did improve dyslipidemia management—but only when combined with robust audit-and-feedback mechanisms [5]. Without such structural support, even well-designed tools fall flat.

*Empowerment: an essential complement—*Thus, the cards alone are insufficient; patient empowerment must be built into implementation. Evidence from adherence research shows that health-literacy–enhancing interventions deliver better outcomes. For example, combining antihypertensive and lipid-lowering medications at once improved six-month adherence [6]. Interventions focusing on health literacy and self-management (e.g., enabling patients to interpret and track their own LDL-C numbers) can shift behavior meaningfully—unlike passive tools.

*Proposal: coupling card with training*—We propose a two-pronged approach:

- Structured patient education at the time of card issuance—teaching how to enter lab values, interpret targets (< 55 mg/dL), and prepare discussion questions for their PCP.
- Automated reminders via SMS or email (at 1, 3, and 6 months) prompting patients to bring updated cards to follow-up visits.

This strategy mirrors the effective model of audit-feedback interventions that yielded improved LDL-C control in primary care. Importantly, it also respects the real constraints on PCP time.

*Call for research and evaluation—*We urge implementation studies to evaluate this model. For instance, cluster-randomized trials could compare standard plus card versus card plus structured education (with and without reminders). Primary outcomes should include:

- Proportion of card reviewed by PCPs
- Patients achieving LDL-C < 55 mg/dL at 6 and 12 months
- Adherence and engagement data (e.g., frequency of card updates, clinic presentation)

Conclusion**—**Lipid cards have the potential to bridge communication gaps and reinforce guideline-based lipid management. Yet, when deployed in isolation, they falter. To fulfill their promise, cards must be embedded within patient-empowerment strategies and supported by system-level enablers (education, reminders, audit loops). Only then can we hope to see meaningful improvements in LDL-C goal attainment, reversing the 75%–25% drop-off, and making a dent in the stark ESC-highlighted treatment gap.
